# Supplementary material for: Hierarchically Porous Silk/Activated-Carbon Composite Fibres for Adsorption and Repellence of Volatile Organic Compounds
Source: Molecules. 2020 Mar 7;25(5):1207. doi: 10.3390/molecules25051207 (PMC7179458; doi:10.3390/molecules25051207)
Supplement: Supplementary file 1 [file molecules-25-01207-s001.zip › molecules-724611-SI.pdf]

molecules

Supporting Information

Hierarchically Porous Silk/Activated-Carbon Composite Fibres for Adsorption and Repellence of Volatile Organic Compounds

Aled D. Roberts, Jet-Sing M. Lee, Adrián Magaz, Martin W. Smith, Michael Dennis, Nigel S. Scrutton and Jonny J. Blaker*

Source materials

Bombyx mori silkworm cocoons were purchased from Wildfibres (Birmingham, UK). Lithium bromide (anhydrous, > 99%), sodium carbonate (anhydrous, > 99%) and poly(ethylene glycol) (10 kDa Mw) were purchased from Sigma Aldrich, UK. The 10 kDa MWCO SnakeSkinTM dialysis tubing was purchased from Thermo Fischer Scientific, UK. Analytical grade ethanol was used for wet-spinning coagulation bath, deionised water and laboratory grade solvents were used routinely. Activated carbon powder was purchased from US Research Nanomaterials Inc; the specified average particle size was 60–80 nm with an SSA of 1350 m^2 g^{-1}, however our analysis revealed the particle size to be ~0.2–5 μm (via SEM) with a BET SSA of ~700 m^2 g^{-1} (via N_2 gas sorption).

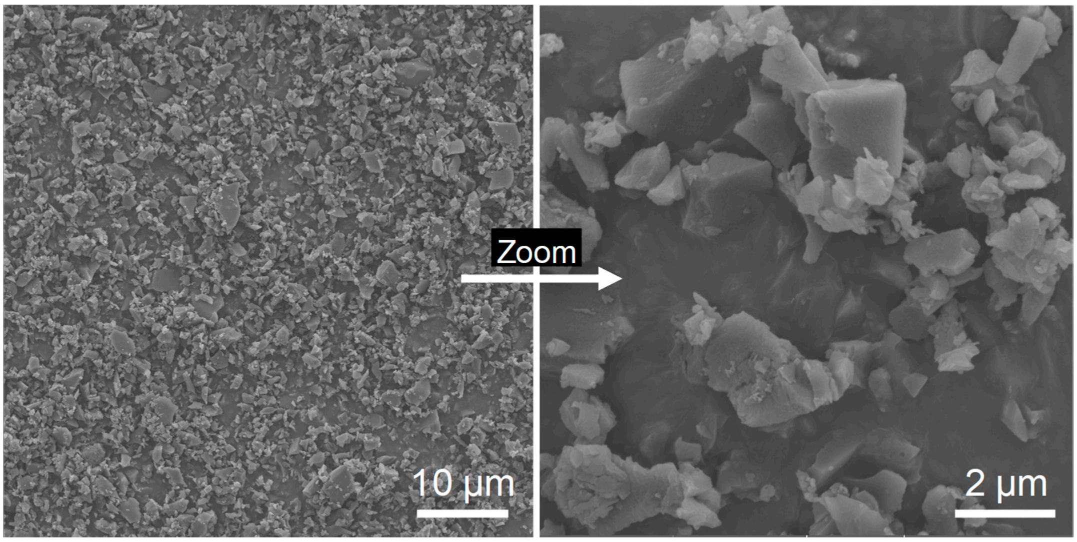

SEM images of the AC powder employed in this study

Characterisation techniques

SEM images were taken using a TESCAN MIRA3 FEG-SEM using secondary electron detection with an accelerating voltage of 5 kV. Samples were adhered to an aluminium stud using conductive carbon tape and sputter coated with a 60:40 Au/Pd alloy (5 nm) prior to imaging. The porous properties of the materials were investigated by nitrogen adsorption and desorption at 77.3 K using a BELSORP-mini (BEL-Japan, Inc.). All samples were degassed at 120 °C for 15 h under dynamic vacuum before analysis. Brunauer–Emmett–Teller (BET) surface area was obtained in the relative pressure (P/P0) range of 0.05–0.20. Cyclohexane adsorption isotherms were measured using a Dynamic Vapour Sorption (DVS) instrument (Surface Measurement Systems Ltd, Alperton, United Kingdom). Samples of fibres (20–50 mg) were loaded into a sample pan, and then heated at 393K for 3 hours in a stream of dry nitrogen. Isotherms were measured by exposing the samples to sequentially increasing concentrations of cyclohexane in a flow of dry nitrogen. At each data point, equilibrium was defined as a rate of mass change of less than 0.001% per minute, maintained below this level for ten minutes. Contact angle measurements were taken using a Kruss FTA100 instrument. Mechanical

data for the fibres was obtained using an Instron 3344 (Instron Ltd., USA) uniaxial tensile testing instrument with a 10 N load cell. Optical and cross-polarised light microscopy images were taken using a Leica TSC SP5 Confocal Microscope. WAXD was performed using a PANalytical X'Pert Pro (UK) instrument with a Cu Kα radiation source, a diffraction angle of 5–60° and scanning rate of 2° min^{-1}. FTIR was performed using a Nicolet iS5 (Thermo Scientific, UK) instrument equipped with an iD5 (diamond) attenuated total reflectance (ATR) attachment; the scan number (per measurement) was 32 with a scan range of 2000–800 cm^{-1} and resolution of 4 cm^{-1}. The protein secondary structure composition was evaluated within the spectrum of the amide I region ranging from 1700 to 1600 cm^{-1} by means of deconvolution analysis.[1]

Experimental details

Preparation of RSF and RSF-AC spinning dopes

RSF spinning dopes were prepared following a previously published protocol with minor modifications.[1,2] Silkworm cocoons were initially de-wormed prior to being degummed by boiling in a 0.02 M aqueous Na_2CO_3 solution for 30 min. The degummed silk fibroin (SF) was then thoroughly washed in DI water before being air-dried for ca. 16 h. The dried and degummed SF was then dissolved in hot (ca. 80 °C) 7.9 M LiBr to a concentration of 16% *w/v*, with gentle stirring at 60 °C for ca. 2 h. The solution was then centrifuged (9000 g, 15 min) to remove residual pupa prior to dialysis (10k MWCO) against DI water (5 L, 4 °C) over 48 h with continuous stirring and regular water changes (at least 6). After dialysis, the solutions were concentrated to the desired spinning-dope concentration via reverse dialysis against aqueous PEG (10 kDa, 15 % *w/v*). The prepared and concentrated RSF spinning dope solutions were stored at 4 °C and used within 72h.

Cryo-SBS spinning protocol

Porous RSF fibres were prepared via cryo-SBS following a protocol previously developed within our group (Fig. 1a).[1,3] Briefly, aqueous RSF and RSF-AC solutions (18–19 w/w % RSF concentration) were extruded through 0.8 mm nozzle via a syringe pump (Aladdin Syringe Pump, WPI Instruments), while a concentric outer nozzle delivers compressed air (typical working pressure 10–60 psi). The laminar flow of air over the meniscus of the extruding spinning dope induces the formation of fibres. The spun fibres were directed into a cryogenic liquid nitrogen (LN2) bath to induce immediate freezing. The typical working distance (WD) from nozzle to LN2 bath was ca. 15 cm. The frozen fibres were then collected and lyophilized (freeze-dried) for at least 24 h, resulting in porous RSF and RSF-AC fibres.

Cryo-WS spinning protocol

Briefly, aqueous RSF and RSF-AC solutions (12–14% w/w % RSF concentration) were extruded into an ethanol coagulation bath through a 21 Gauge 40 mm needle via a syringe pump at a rate of 100 μL min^{-1} (Fig. 1b, Videos S1 and S2). The spun fibres were collected on a rotating reel and prevented from drying out through occasional rinsing with EtOH. The collected fibres were then submerged in an excess of DI water and allowed to equilibrate for approximately 5 min before the water was refreshed. This was repeated a total of 3 times to ensure adequate solvent exchange (EtOH exchanged for DI water). The hydrated fibres were then submerged in LN2 until completely frozen, before being collected and lyophilized (freeze-dried) for at least 24h, resulting in porous RSF and RSF-AC fibres.

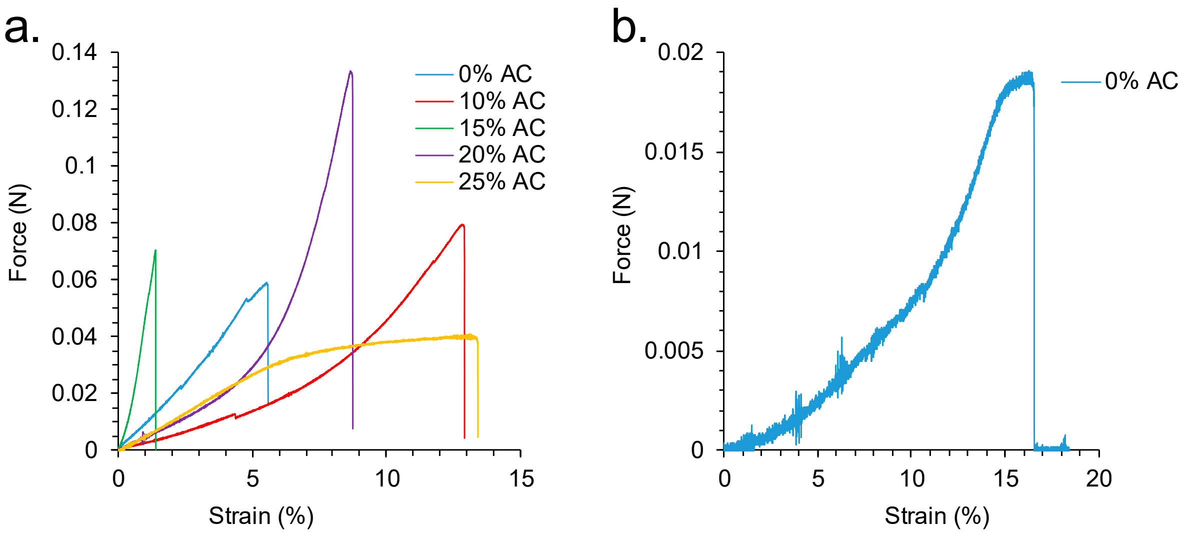

Figure S1. Uniaxial force-strain curves for (**a**) RSF and RSF/AC fibres produced by Cryo-WS, and (**b**) RSF fibre mats produced by Cryo-SBS.

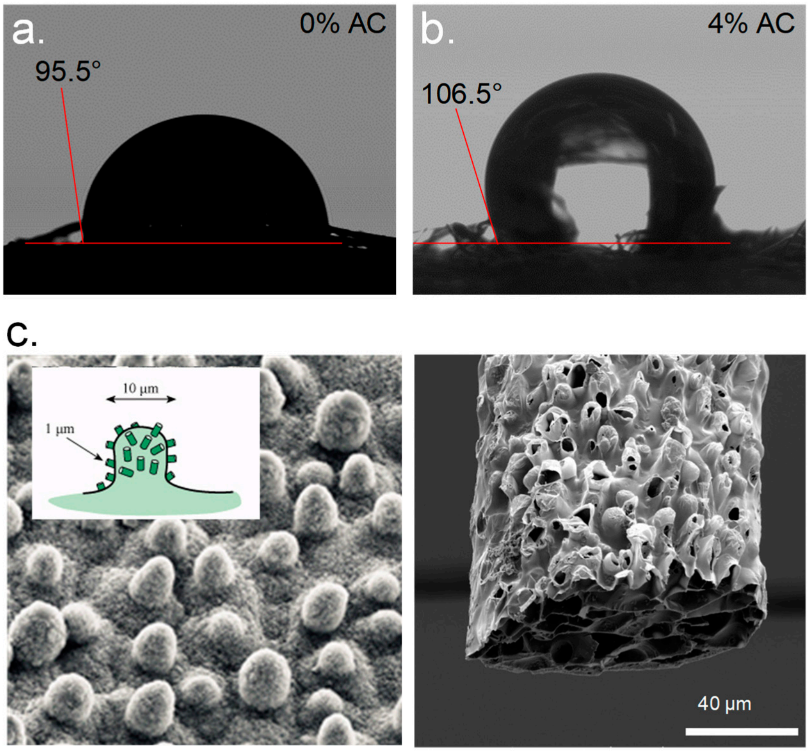

Figure S2. Contact angle measurements for RSF fibres produced by Cryo-SBS with (**a**) 0% and (**b**) 4% AC loading. (**c**) Comparison between superhydrophobic lotus leaf surface structure[4] (left) and a Cryo-SBS fibre (right).

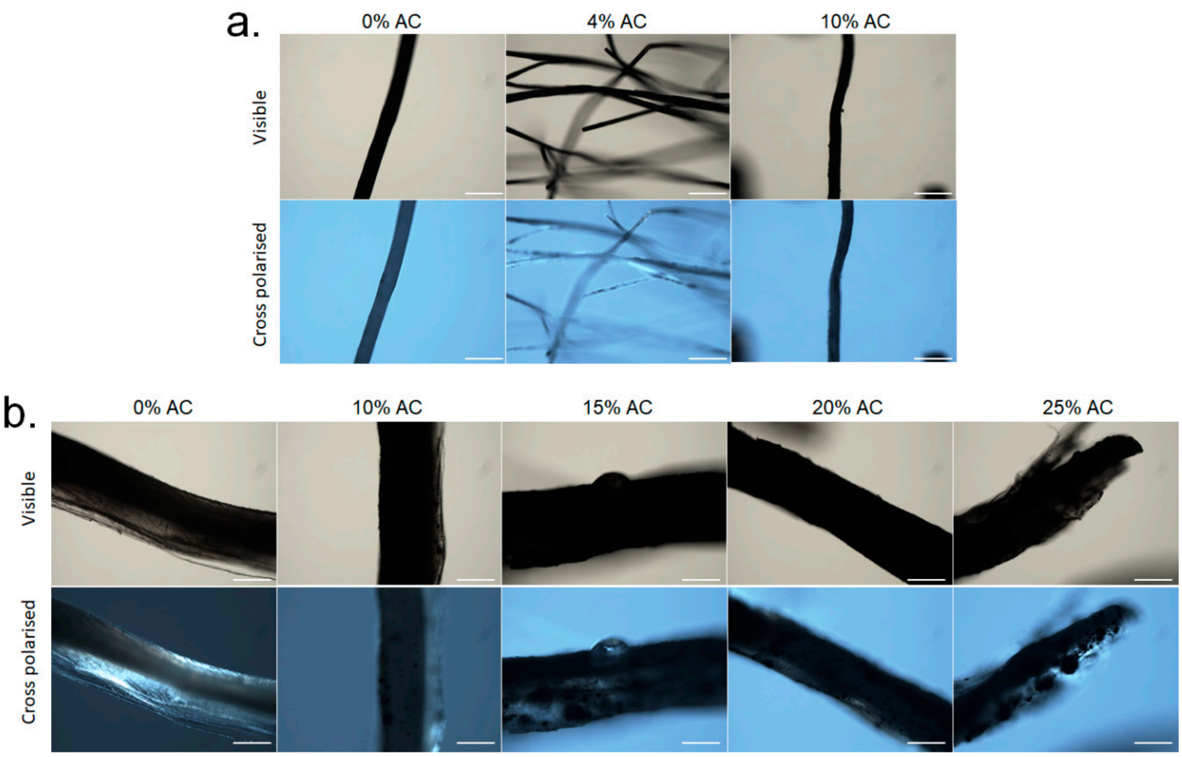

Figure S3. Visible (above) and cross-polarised (below) light microscopy images of porous RSF and RSF/AC fibres produced by (**a**) Cryo-SBS and (**b**) Cryo-WS techniques. Scale bars = 200 μm.

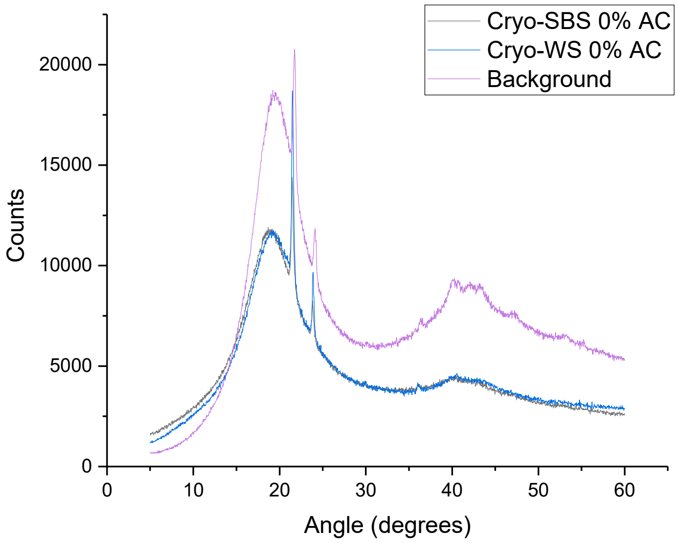

Figure S4. WAXD patterns for porous RSF fibres produced via Cryo-SBS and Cryo-WS methods. A background sample was included for comparison; all peaks were attributed to the substance adhering the fibres to the sample holder (i.e., petroleum jelly).

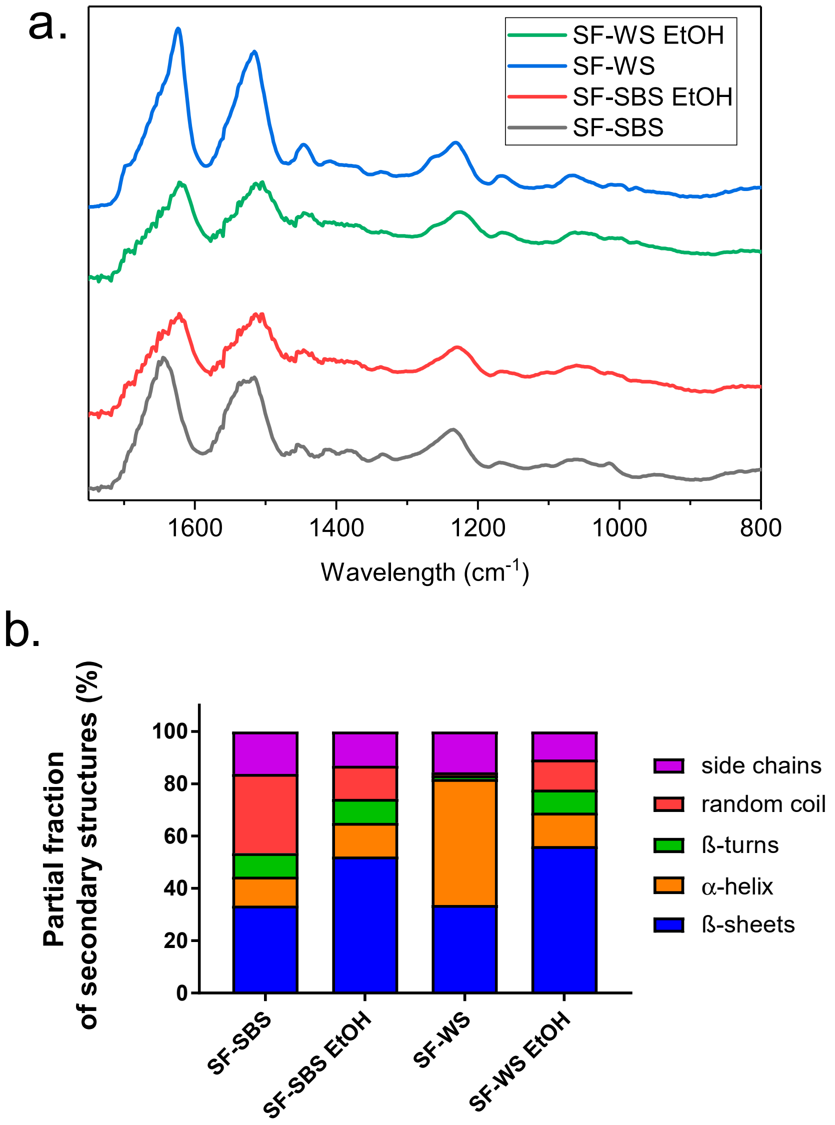

Figure S5. (**a**) Normalized ATR-FTIR spectra of RSF fibres produced through Cryo-SBS and Cryo-WS techniques before and after EtOH treatment. (**b**) Secondary structure composition assessed by deconvolution of the amide I regions of the above spectra.

References

1. A. Magaz, A. D. Roberts, S. Faraji, T. R. L. Nascimento, E. S. Medeiros, W. Zhang, R. D. Greenhalgh, A. Mautner, X. Li, and J. J. Blaker: Porous, Aligned, and Biomimetic Fibers of Regenerated Silk Fibroin Produced by Solution Blow Spinning. *Biomacromolecules* **19**(12), 4542 (**2018**).
2. D. N. Rockwood, R. C. Preda, T. Yücel, X. Wang, M. L. Lovett, and D. L. Kaplan: Materials fabrication from Bombyx mori silk fibroin. *Nat. Protoc.* **6**(10), 1612 (**2011**).
3. E. L. G. Medeiros, A. L. Braz, I. J. Porto, A. Menner, A. Bismarck, A. R. Boccaccini, W. C. Lepry, S. N. Nazhat, E. S. Medeiros, and J. J. Blaker: Porous Bioactive Nanofibers via Cryogenic Solution Blow Spinning and Their Formation into 3D Macroporous Scaffolds. *ACS Biomater. Sci. Eng.* **2**(9), 1442 (**2016**).
4. H. J. Ensikat, P. Ditsche-Kuru, C. Neinhuis, and W. Barthlott: Superhydrophobicity in perfection: the outstanding properties of the lotus leaf. *Beilstein J. Nanotechnol.* **2**(1), 152 (**2011**).
